# Nutrition and IBD: Malnutrition and/or Sarcopenia? A Practical Guide

**DOI:** 10.1155/2017/8646495

**Published:** 2017-01-03

**Authors:** F. Scaldaferri, M. Pizzoferrato, L. R. Lopetuso, T. Musca, F. Ingravalle, L. L. Sicignano, M. Mentella, G. Miggiano, M. C. Mele, E. Gaetani, C. Graziani, V. Petito, G. Cammarota, E. Marzetti, A. Martone, F. Landi, A. Gasbarrini

**Affiliations:** ^1^Gastroenterology Division, Catholic University of Sacred Heart, Rome, Italy; ^2^Nutrition Team, Catholic University of Sacred Heart, Rome, Italy; ^3^Gerontology Division, Catholic University of Sacred Heart, Rome, Italy

## Abstract

Malnutrition is a major complication of inflammatory bowel disease (IBD). This mini review is focusing on main determinants of malnutrition in IBD, the most important components of malnutrition, including lean mass loss and sarcopenia, as an emerging problem. Each one of these components needs to be well considered in a correct nutritional evaluation of an IBD patient in order to build a correct multidisciplinary approach. The review is then focusing on possible instrumental and clinical armamentarium for the nutritional evaluation.

## 1. Introduction

Malnutrition is a major complication of inflammatory bowel disease (IBD) and it is primarily responsible for chronic weight loss. Existing data suggest that malnutrition affects a large portion of patients with inflammatory bowel disease (IBD), estimated in 65–75% of patients with Crohn's disease (CD) and in 18–62% of patients with ulcerative colitis (UC). Based on BMI analysis, malnutrition prevalence seems to be higher in CD compared to UC, although several authors reported similar prevalence of malnutrition in both conditions [1–4]. In pediatric patients, malnutrition is main cause of growth retardation and it could anticipate gastrointestinal symptoms. Prevalence of malnutrition in pediatric patients seems to be higher in CD patients than in UC patients [5, 6].

Impairment of nutritional status has a multifactorial etiology. Main reasons include suboptimal energy intake, malabsorption, enteric nutrient loss, increased basal energy expenditure, and medications. In clinical settings, IBD patients are commonly found to be underweight, with several nutritional deficiencies, alterations of anthropometric parameters, in body composition of fat, and muscle mass and with low bone mineral density [2, 3, 7].

## 2. Main Determinants of Malnutrition in IBD

The* decrease of oral food intake* is one of the most important reasons for malnutrition in patient with IBD. Two main mechanisms are reported. The first is connected to the disease itself: patients avoid eating due to symptoms such as nausea, abdominal pain, vomiting, and diarrhea during disease inflammatory activity. The second is connected to fasting due to hospitalization or prolonged restrictive diets [8, 9]. Moreover, energy intake is also linked to disease localization in CD patients, with a reduction of energy intake only in ileal and ileocolonic disease [10].

Existing data correlate malabsorption* to inflammatory cytokines* released from immune cells within gut mucosa of CD and UC patients during active and remission phases. Malabsorption mechanisms are related to epithelial alterations such as impaired epithelial transport and loss of epithelial integrity. Other important mechanisms seem to be bacterial overgrowth and increased intestinal mobility [11, 12]. Malabsorption seems to play a major role in patients with BMI less than 18.5 kg/m^2^ [4, 13].

*Gastrointestinal nutrient loss* is strictly connected to malabsorption and is caused by both epithelial alteration and disease activity, which mainly lead to loss in nutrient and in active phases of disease by inflammatory diarrhea. In particular, alterations of ionic transports cause dispersion of electrolytes and water in intestinal lumen. Inflammation can also lead to the ulceration of the bowel surfaces, which in turn determine chronic blood loss and proteins loss within the intestinal lumen. A correct management of IBD patients can prevent anemia and hypoalbuminemia [9, 11, 14].

Another important mechanism is* the biliary salt diarrhea*, usually associated with a terminal ileal disease, with an impaired absorption of fat-soluble vitamin and lipids. Fat malabsorption can lead to steatorrhea [15].

Several studies investigated the increased* energy expenditure* in IBD. There are two key points: the first is the comparison of energy requirement among CD and UC patients and controls and the second is the difference between carbohydrate metabolism and lipid metabolism.

The energy requirement in IBD patient can be valued with the Harris-Benedict formula for basal energy expenditure (BEE) or with indirect calorimetric measurements of resting energy expenditure (REE). BEE represents the energy expenditure required for the maintenance of vital functions at rest and during the fasting; REE, instead, represents the energy expenditure during nonfasting time, including BEE and the thermic effect of food. No significant differences were found in energy requirement among IBD patients and controls using BEE or REE [16]. However, there is a significant difference in basal energy expenditure between IBD patients with a regular body weight and IBD patients with reduced body weight. For instance, patients with body weight less than 90% of the ideal body weight have higher energy expenditure than patients with body weight more than 90% of the ideal value, estimated in up to 24% of energy expenditure per kilogram over the control patients [7, 16].

Other important evidences suggest major differences in carbohydrate oxidation and lipid oxidation in IBD. In particular, CD patients show higher lipid oxidation and lower carbohydrate oxidation rate in basal condition compared to UC patients and controls. UC patients show a little increase in lipid metabolism and similar carbohydrate utilization compared to controls [2, 10]. In children with IBD, the energy intake and metabolism rate are variable according to age and weight of each patient [5, 6].

*Medications* could have an impact on micronutrient absorption and utilization.

Glucocorticoids can interfere with calcium, phosphorus, and zinc absorption and utilization. They are also related to an impaired metabolism of vitamins C and D. Long-term glucocorticoid exposure is associated with bone alteration and osteoporosis.

Sulfasalazine is a folic acid antagonist and long-term therapies are related to anemia and hyperhomocysteinemia.

Cholestyramine can interfere with absorption of fat-soluble vitamins, iron, and B_12_ vitamin. Main side effect is steatorrhea due to impairment absorption of fats.

Moreover, the use of long-term parenteral nutrition can lead to micronutrient deficiencies, including vitamins A, D, and E, zinc, copper, and selenium [1, 15].

**Figure 1:**
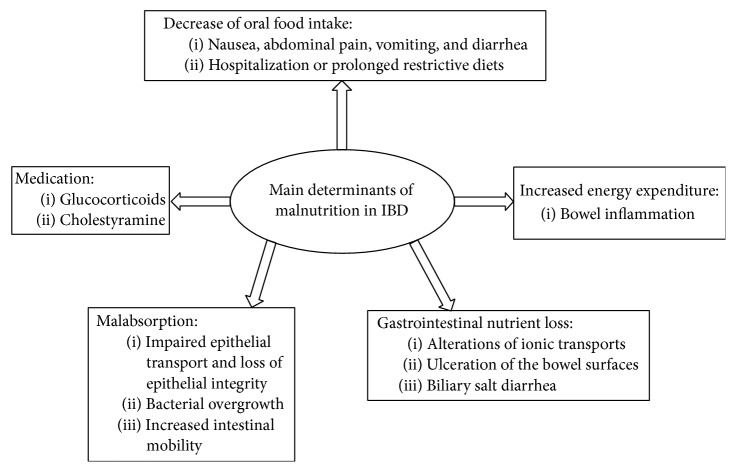
Main determinants of malnutrition in IBD.

Main determinants of malnutrition in inflammatory bowel disease are summarized in Figure 1.

## 3. Clinical Aspects of Malnutrition in Patients with IBD

*Micronutrient and vitamin deficiencies* are common in IBD patients and preventions of those deficiencies are important to avoid clinical complications. Types of deficit depend on many factors as disease localization and extension, disease activity, alimentation, nutritional support, and medication used for IBD (Table 1).

The most common micronutrient deficiencies in IBD are iron, calcium, selenium, zinc, and magnesium depletion. Vitamin deficiencies include all vitamins and in particular B_12_, folic acid (water soluble), and vitamins A, D, and K (fat-soluble) [1, 15].

Selenium, zinc, and magnesium depletions are caused by inadequate dietary intake and chronic loss for diarrhea. The exact prevalence in IBD is still unclear. Symptoms associated with deficiencies include bone health impairment, fatigue, poor wound healing, and cartilage degeneration [1, 15].

Several studies documented vitamin A deficiency in up to 90% of IBD patients as a result of inadequate intake, low BMI and low fat mass and ileal location of disease or bowel resection, ileal resection, disease duration, and higher CRP level. Vitamin A deficiency associates with poor wound healing, night blindness, and xerophthalmia [1, 15].

Vitamin K deficiency is also reported in IBD patients, with unknown prevalence. Main determinants of vitamin K deficiency are disease activity, use of antibiotics, and malabsorption, as the most important source of vitamin K is intestinal production by gut microbiota. Vitamin K deficiency contributes to alteration of coagulation factors and may contribute also to abnormal bone metabolism [1, 15].

*Anemia* is another clinical aspect of malnutrition in IBD; its prevalence covers up to 70% of pediatric patients and up to 50% of adult patients [17]. Anemia is usually associated with other important symptoms like fatigue, sleeping disorders, restless legs syndrome, attention deficit, discontentment, agitation, or female infertility. Diagnostic criteria are not different from anemia in other types of patients. Main causes of anemia include iron deficiency, B_12_ vitamin and folic acid deficiencies, bleeding from mucosal lesions, surgery, systemic inflammation, and medications. Iron deficiency is the most frequent cause of anemia in IBD patients, like in healthy people, with prevalence estimated in 36–90% [17]. It was demonstrated that iron deficiency has multifactorial etiology, including inadequate intake, blood loss from damaged intestinal wall, impairment absorption (disease localization in duodenum or proximal tract of jejunum), and impairment utilization (due to the systemic inflammatory status) [18, 19]. Furthermore, iron absorption and metabolism are regulated by Hepcidin and soluble transferrin receptors, the first being an important hormone depressing iron absorption and iron release from body stores in iron-overload and in inflammatory status; the second, conversely, increases during iron deficiency status and increases intestinal absorption and release from stores. In active IBD, Hepcidin takes control of iron metabolism with development of anemia due to relative or absolute iron deficiency [20].

Main causes of non-iron deficiency anemia in IBD are anemia of chronic disease (ACD) and deficiencies of vitamin B_12_ and folic acid (vitamin B_9_). ACD is perhaps the most frequent cause of anemia in hospitalized patients [17]. B_12_ deficiency is common in IBD patients, with estimated prevalence of 22% in CD patients and up to 3% in UC patients [1, 21]. B_12_ can be absorbed only in terminal ileal tract due to a complicated mechanism of absorption and all CD patients with ileal resection greater than 60 cm will develop B_12_ deficiency. It is associated with megaloblastic anemia and peripheral neuropathy. Low level of vitamin B_12_ can lead to hyperhomocysteinemia, which is an independent risk factor for venous thrombosis [22, 23].

Folic acid deficiency is more common in IBD patients and it is reported in 28.8% of the CD patients and 8.8% of ulcerative colitis (UC) patients [22]. Risk factors for deficiency include low dietary intake, active disease, malabsorption, and interaction with medicines as sulfasalazine or methotrexate treatment [22–24].

*Low levels of calcium and vitamin D* are common in patients with IBD, especially in those with duodenal and jejunal disease [1, 15]. Calcium deficiency is linked to vitamin D deficiency, because its absorption is governed by vitamin D levels, which can activate the calcium transporters. Vitamin D deficiency is related to inadequate daily intake, inflammation status, diarrhea, and glucocorticoid therapies. The prevalence among IBD patients is up to 70% in CD patients and up to 40% in UC patients. Vitamin D levels are also correlated to disease activity and existing data suggest that vitamin D could be a risk factor for IBD. Vitamin D is implicated in preserving mucosal integrity and mucosal healing capacity and its deficiency may compromise mucosal barrier, increasing risk for mucosal damage and for IBD. In particular, high levels of active vitamin D seem to reduce the risk of developing CD, while not changing significantly the risk for UC [1, 15, 25, 26].

Another significant aspect of malnutrition in IBD is linked to* alteration of body composition*, especially in alteration between fat mass (FM) and fat-free mass (FFM). FM consists of adipose tissues (both visceral and subcutaneous); instead FFM is the body portion which excludes adipose tissue and consists of water, proteins, and mineral and other components [3]. Although there are few small and heterogeneous studies that analyze the role of body composition alteration in IBD patients, it was demonstrated that one-third of CD patients had a significant reduction of body mass index, but only 5% were underweight by BMI criteria. Using second-line methods, it was demonstrated that 28% of CD patients had reduced FFM and 31% of these patients had a significant reduction in FM. Similar data were found in UC patients, in which significant BMI reduction was reported in 20% and a depletion of FFM and FM in 13%, while no one of UC patients was underweight by BMI criteria. These data suggest that BMI variations are not strictly connected to alteration in body compositions; in particular, there is no connection between FFM and FM depletion and BMI [27]. Conflicting data exist about the exact role of disease activity in body composition; several studies fail in finding connection between disease phase and alteration of BMI, FM, and FFM; others have shown a significant difference in these parameters; in particular, there is an important reduction of BMI and FM in active phase in CD patients, while in remission phase there is also FFM depletion. UC patients have a significant FM reduction in active phase compared to remission phase, but differences between active and remission phases were not significant for BMI and FFM [3]. Moreover, it was reported that FM depletion which occurred in active phase was partially recovered during remission phase instead of FFM which remained depleted also in remission phase.

## 4. The Deficit of Lean Mass in IBD and Sarcopenia: Emerging Aspects

Sarcopenia is a syndrome characterized by progressive and generalized loss of skeletal muscle mass and strength with risk of poor quality of life and physical disability. Principal mechanisms involved in genesis of sarcopenia are malnutrition, immobility, low protein synthesis, and increased proteolysis [28, 29]. Diagnosis of sarcopenia comprehends the demonstrations of both muscle mass and muscle strength. The gold standard to asses muscle mass is DXA, computed tomography (CT), or magnetic resonance imaging (MRI), but they have a limited use in clinical practice due to high costs. BIA can be a valid alternative to DXA due to lower cost instead of anthropometric measures. Muscle strength can be assessed with a grip strength test, which is of low cost and is an easy-to-handle technique, using a standard dynamometer [28]. Recent evidences suggest that prevalence of sarcopenia is up to 12% in CD patients evaluated with the appendicular skeletal muscle index (ASMI) asserted with whole-body DXA. Moreover, a multivariate analysis found that grip strength test was a positive predictor of low ASMI compared to BMI [30].

Consequences of sarcopenia are bone demineralization and pathological fractures, cardiovascular disease and higher probability of hospitalization, and reduction of mobility [29]. Furthermore, several studies associated sarcopenia with osteopenia: osteopenia has been evaluated as reduction of bone mineral density (BMD) with DXA and *t*-score analysis. Osteopenia was evident in 30% of CD patients in Bryant et al.'s study and in 36% of UC patients. Low lean mass, sarcopenia, and low BMI are all independent positive predictors for osteopenia and osteoporosis [30].

A correlation between IBD and obesity has also been described. The prevalence of obesity is quite different across different studies and likely this depends on obesity prevalence in general population. Up to 32.7% of IBD patients are obese, and in particular up to 30.3% of CD patients and up to 35.2% of UC patients [27, 31]. Obesity is associated with more anal and perianal complication [32]. Moreover, overweight patients had a better clinical course than normal weight patients. Obesity did not increase healthcare hospitalization and surgery operations. Clearly, more studies are needed to individuate the optimal therapy for IBD obese patients [33].

Two other teams investigated the influence of obesity on anti-TNF-alfa treatment with contrast results. Flores et al. found that IBD patients with BMI > 25 kg/m^2^ are less likely to need anti-TNF therapy than normal or underweight patients and are less likely to need surgery or hospitalization. Overall, they define obesity as a marker of less severe disease [31]. Instead, Harper et al. reported that high BMI in IBD patients is associated with a greater need for Infliximab escalation dose and with higher loss of response, need for steroid therapy, and number of hospitalization days compared to normal BMI patients [34]. The reasons appear to be unclear and further investigations are needed.

Sarcopenia and obesity in IBD patients can lead to sarcopenic obesity. Sarcopenic obesity is defined in literature as a clinical condition in which criteria for sarcopenia met criteria for obesity; this syndrome is not simply the sum of the two conditions. Obesity and sarcopenia are both responsible of physical impairment and metabolic disorders and may act with each other. Sarcopenic obesity is related to a fast functional decline of patient's status, with a high risk of disability, mobility, and mortality; for these reasons diagnosis is important. Screen test can be simply grip strength test and BMI or otherwise body composition study with DXA [29].

## 5. Pathophysiology of Malnutrition and Sarcopenia in Inflammatory Bowel Disease

**Figure 2:**
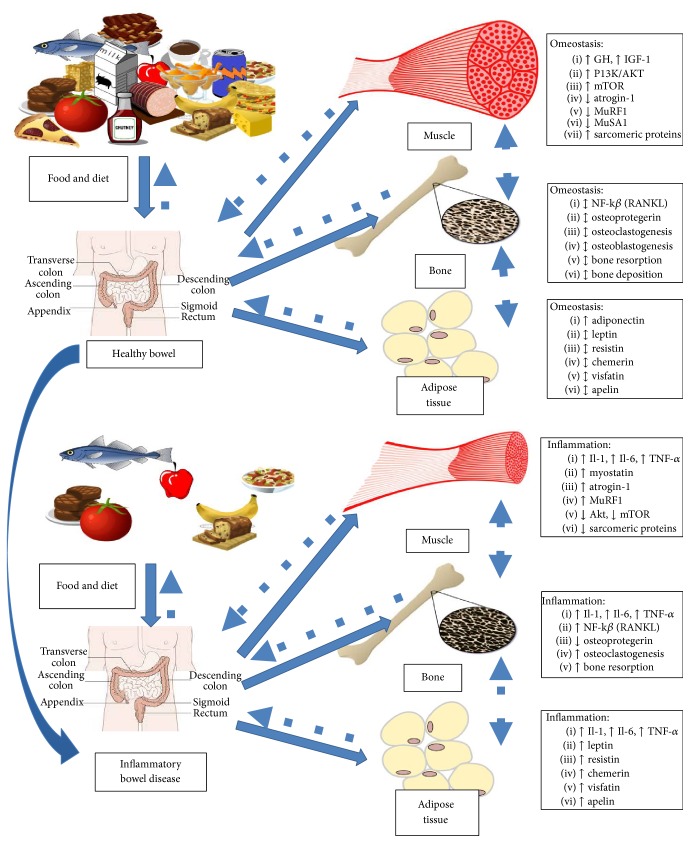
Pathophysiology of malnutrition and sarcopenia in inflammatory bowel disease. Interplay between nutrition, inflammation, muscle, bone, and adipose tissue in healthy subject and IBD.

In healthy subjects, nutritional status and body composition are maintained by complex interactions between muscle, bone, and adipose tissue. This perfect balance permits the normal growth of body and the regulation of energy metabolism; moreover, it is crucial for the environmental adaptation of organism. Each factor of this balance is able to “communicate” with the others through a complex cell-signaling network, influencing the activation and the development of each other (Figure 2). Many pathological conditions can alter this balance, causing deregulation of energy metabolism and body composition, with hyperexpression and reduction of particular tissues. Inflammatory chronic diseases, infections, liver cirrhosis, and heart and kidney failure are the most diffuse diseases that are able to influence the nutritional status and the body composition through the deregulation of metabolism of muscle, bone, and adipose tissues.

In particular, inflammatory bowel diseases and other conditions characterized by chronic inflammation can alter the homeostasis of muscles, bones, and fat and are involved in the pathogenesis of malnutrition and dysregulated body composition (Figure 2). Therefore, it is crucial to analyze the role of inflammation for each factor involved in maintaining energy balance [35–37].

Skeletal muscle is the most diffuse tissue in human body and it is involved in many physiological functions like the uptake and the metabolism of glucose. Muscles are regulated by physiological stimuli and pathological conditions that influence size and mass through the control of protein turnover. In chronic inflammation, a shift of protein turnover poised toward protein degradation with subsequent reduction of myofibrillar proteins can be observed, resulting in loss of muscle mass and impaired muscular contraction. In inflammatory chronic diseases, muscle protein degradation seems to depend on a coordinated network of signaling pathways regulated by hormones and cytokines, which reduce synthesis and increase protein degradation. Current evidences indicate that several cellular pathways, involved in cellular growth, are deregulated during chronic inflammatory disorders, in particular the ubiquitin proteasome and the autophagy pathways. The most important pathway involved in muscle growth is the insulin/GH/IGF1 system. Normally, circulating GH binds to transmembrane GH receptors, activating the synthesis of IGF1 in liver and other tissues as bone and muscles. In bones, IGF1 stimulates the proliferation of chondrocytes and osteoblasts in the epiphysis of long bones, resulting in linear growth, while in muscular cells IGF1 enhances protein synthesis via P13K/AKT phosphorylation with activation of mTOR system and reduction of activation of ubiquitin ligands [38]. In IBD, as in chronic inflammatory conditions, a significant reduction in plasma and muscle IGF1 can be seen in response to the elevated concentrations of TNF-alpha and IL-6, cytokines that cause GH resistance in liver and muscles, inducing downregulation of mTOR pathway with activation of ubiquitin ligands and expression of enzymes involved in protein degradation, in particular atrogin-1, MuRF1, and MUSA1 [39–41]. On the contrary, during chronic inflammation, a significant activation of myostatin, a member of TGF-beta family, can be observed. Myostatin is able to induce degradation of sarcomeric proteins through the synthesis of atrogin-1 and MuRF1 via the upregulation of the transcription factors Smad2/3 and the suppression of Akt signaling [42, 43]. Moreover, in chronic inflammatory conditions, acute phase proteins and cytokines as TNF-alpha, IFN-gamma, and IL-6 exert a direct role in muscle wasting, in particular by activating the transcription factor NF-kB and the ubiquitin proteasome system and interfering with the myogenic program. For example, TNF-alpha can impair proliferation and differentiation of muscular steam cells, acting on NF-kB experimental model [44].

Viewed only as passive energy reservoir in past, it is now clear that adipose tissue is an important and active endocrine organ, involved in the modulation of energy metabolism and in the bone growth [45]. Adipose tissue has also a significant immunological role, in particular modulating the immune response via recruitment and activation of immune cells and differentiation of lymphocyte [46]. Dysregulation of adipose tissue is frequent in chronic conditions like diabetes, hypertension, and heart diseases, conditions which are all generally characterized by hyperexpression of fat tissue and low grade of inflammation.

In IBD, more in Crohn's disease, a significant increase in visceral adiposity can be observed, in particular in mesenteric fat. Moreover, the presence of an enlarged mesenteric adipose tissue that envelopes more than a half of the intestinal circumference in corresponding inflammatory lesions is reported. This type of adipose tissue, known as “creeping” fat, does not depend on BMI and it is an important indicator of disease activity, correlating with transmural inflammation and its complications (strictures and fistulas) [47, 48]. Mesenteric fat seems to be involved in the pathogenesis of Crohn's disease: adipocytes in mesenteric fat can produce inflammatory cytokines such as TNF-alpha, IFN-gamma, IL-6, and IL-1 and other adipokines as leptin and resistin. These molecules demonstrate a strong proinflammatory effect, activating immune response and deregulating intestinal homeostasis [36]. Moreover, it is now clear that adipocyte mass correlates with the degree of expression of these cytokines and disease activity [49].

In addition, adipose tissue can express several inflammatory adipokines as leptin. This adipokine is overexpressed in mesenteric fat in patients with active Crohn's disease compared with controls and it has significant proinflammatory activity [50]. Leptin indeed has structural and functional similarities with IL-6; it can stimulate the proliferation of blood mononuclear cells, increase the proliferation and the survival of CD4 T cells, stimulate the development of Th1 response, and promote dendritic cell differentiation via the activation of intracellular JAK-STAT pathways [51, 52]. Another adipokine hyperexpressed in mesenteric fat in Crohn's disease is resistin. Resistin is inducted by bacterial lipopolysaccharides and enhances the secretion of TNF-alpha and IL-12 in humans and the activation of mononuclear cells [53–55]. Other adipokines hyperexpressed in IBD seem to be visfatin and apelin which have significant proinflammatory activity, while the exact role of adiponectin is not clear yet [56, 57].

Skeletal homeostasis is a complex system, influenced by a delicate balance between bone formation and reabsorption, a process called bone remodeling. This delicate process is coordinated by a number of factors, severely impaired in patients with IBD. Although the pathogenic mechanism of bone loss in IBD has a multifactorial etiology, it is now clear that chronic inflammation plays a crucial role through inflammatory mediators like TNF-alpha, IL-6, and IL-1. In particular, these cytokines dysregulate the receptor activator of nuclear factor *κ*B ligand (RANKL) osteoprotegerin (OPG) system: normally RANKL is a strong activator of osteoclastogenesis, while OPG is linked with osteoblastogenesis; thus the interaction of RANK on the surface of osteoclasts with its ligand RANKL induces osteoclastogenesis and conversely the interaction with the osteoblast derived soluble decoy receptor; osteoprotegerin (OPG) blocks RANK interaction inhibiting osteoclasts formation [58, 59]. In experimental models, proinflammatory cytokines induce RANKL and promote bone reabsorption with consecutive bone loss; moreover, activated T cells can directly trigger osteoclastogenesis through RANKL leading to bone [60].

Furthermore, it is now clear that bone metabolism is directly influenced by adipose tissue. Adipokines, indeed, seem to interfere in bone metabolism by altering the sensitive balance between osteoblasts and osteoclasts: in particular, resistin seems to increase the number of differentiated osteoclasts and stimulate the NF-kB promoter activity; when administered in vivo [61], adiponectin has a negative correlation with bone mass in vivo [62].

## 6. Nutritional Assessment of Inflammatory Bowel Disease

The assessment of nutritional status in IBD is the first step to identify malnutrition and/or sarcopenia status in order to set adequate and customized therapeutic regimens.

A correct nutritional assessment provides quantitative and qualitative evaluations, so that effectiveness of nutritional support could be measured and evaluated over time.

An accurate assessment of nutritional status could account for differential methods and diagnostic indicators, as a unique laboratory or instrumental marker is not available.

Main steps in a full evaluation require assessment of the energy balance, assessing anthropometric data, considering biochemical and clinical evaluation, and use of instrumental data.

The assessment of the energy balance is needed to establish the proper nutritional intervention and can be calculated on the basis of caloric intake and energy expenditure of patients with IBD. The caloric intake can be estimated by collecting food chart on eating habits (quantity and quality). Although the best estimation on energy expenditure can be measured by indirect calorimetry or predictive formulas, in current clinical practice, the formula of Harris-Benedict (rated basal metabolism or BEE) in combination with the possible factors of stress (TEE Kcal/day = BEE × stress factor) could be considered [63].

The evaluation of body composition needs to consider weight, height, BMI (body mass index), and body circumferences.

The body circumferences are indicators of the transverse dimensions of the body segments and of the muscle-fat areas of limbs; these parameters could be useful in patients with IBD to estimate nutritional status. Arm's and calf circumferences are the most useful indicators for the evaluation of energy reserves and muscle mass, also included in the evaluation test for screening malnutrition, known as MNA test.

BMI values <18.5 kg/m^2^ are considered indicators of malnutrition [64], whereas values >25 kg/m^2^ indicate overweight; patients with a BMI over >30 kg/m^2^ are considered obese, while persons with a value inferior to 14-15 kg/m^2^ of BMI are at risk for increased mortality due to malnutrition.

A full characterization of the body composition could be reached by the determination of lean body mass and adipose tissue, using specialized machineries, including dual-energy X-ray absorptiometry (DEXA), Bioimpedentiometry (BIA), TOBEC (Total Body Electrical Conductivity), ultrasound, CT (computed tomography), infrared interactance, and NMR (Nuclear Magnetic Resonance).

Among these, BIA and DEXA are considered the gold standard for measuring body composition [65].

DEXA is based on the principle of X-ray attenuation administered at two different energy levels; according to the intensity of the radiation registered after the passage through the tissues, the bone tissue can be differentiated from the soft parts, such as adipose tissue and muscle. The DEXA gives information on bone mineralization.

The BIA is an indirect technique for measuring body composition based on two physical principles: resistance (Rz) and reactance.

The resistance is the ability of all biological structures to “resist” (to counteract) the passage of electricity and it is inversely proportional to the water content: lean tissues are good conductors (containing water and electrolytes), while fatty tissues and bones are poor conductors (containing little water).

The reactance (Xc) is the opposing force to electricity measured at cell membranes levels; fat mass has low reactance, while lean body mass has high reactance.

The combined analysis of the two data allows us to calculate a new parameter, called* “phase angle.”* In healthy subjects, normal values are considered between 6° and 8°; increased catabolic conditions like sarcopenia associated with decrease in these values below 6, while phase angle increases for muscle hypertrophy. Approximately, values of the phase angle under 4,5° indicate a possible expansion of the extracellular space and loss of cell membranes in forms of protein-energy malnutrition.

Among other methods, TOBEC (Total Body Electrical Conductivity) needs further explanation. It is based on the difference of electrical conductivity between lean and fat tissues. The electricity generates a weak magnetic field in a cylindrical chamber whose strength is related to the body composition. In about 10 seconds, TOBEC estimated lean body mass. It accounts for good accuracy, safety, and reliability; however, it cannot be used in case of major changes in weight or distributions of water or electrolytes [66].

The use of ultrasound on lean mass evaluation is limited, as it is strongly dependent on the operator [67].

Infrared interactance, based on the principle of absorption and reflection of spectroscopic light, has good potential but its application is limited [66].

CT is a technique that uses ionizing radiation to examine sections or layers body; despite its high accuracy, cost and radiation exposure do not justify its use in clinical practice.

The NMR (Nuclear Magnetic Resonance) is based on the principle of magnetic fields. NMR can be used for evaluation of the amount of total fat mass and analysis of the regional distribution of adipose tissue. The high cost of the instrumentation and the low practicality do not justify its use in the common practice.

Tables and nutritional questionnaires have been proposed to standardize the nutritional evaluation, assessing several items by numerical scores. Thanks to that, a degree of “the nutritional risk” associated with malnutrition is provided in order to influence the choice of the nutritional intervention.

The main screening tests for malnutrition are the following [68]:

- Instant Nutritional Assessment (INA)
- Malnutrition Universal Screening Tool (MUST)
- Nutritional Risk Screening (NRS)
- Mini Nutritional Assessment (MNA)
- Subjective Global Assessment (SGA)

INA, also called LAW (acronym coming from the first letters of each studied parameter), is based on three parameters: the count of lymphocytes in the blood, serum albumin, and the weight change per unit of time.

MUST, developed in the UK by the Malnutrition Advisory Group, is designed to identify patients at risk of malnutrition, which could benefit from nutritional intervention; it was first developed for community medicine but it had good success on hospitals evaluations because of its simplicity, reliability, and validity [69]. The nutritional risk is assessed with the identification of weight and height to calculate BMI, assessment of unintentional weight loss in the last 3–6 months, and the presence of any acute medical condition, in which there was an insufficient food intake calculated for a period equal to or greater than 5 days. The total score ranges from 0 to 2, indicating the presence of a mild, moderate, or severe risk of malnutrition (low risk = 0, moderate risk = 1, and severe risk = 2) [70].

NRS is commonly used for hospitalized patients [71]. It starts by same parameters of MUST, adding the degree of severity of diseases and age.

NRS 2002 is divided into two parts: an initial screening consisting in 4 questions (BMI below 20.5, weight loss in the last three months, reduced food intake in the last week, and the presence of severe acute disease) and a second part consisting in the evaluation of degree of malnutrition associated with the severity of the disease and age. The second part of the questionnaire is completed if at least one of the four initial questions is positive; a score higher than 3 identifies patients at higher nutritional risk. These last patients require stronger therapeutic intervention.

MNA is a screening test for malnutrition mainly used in elderly people. It includes the assessments of general evaluation (lifestyle, physical activity, and medication), food chart analysis (number of meals, dysphagia, and autonomy), subjective symptoms (patient's perception of the health and nutrition), and detection of anthropometric parameters (reduction weight, BMI, circumference, and skinfolds). A total score below 17 is indicative of malnutrition, while a score between 17 and 23.5 is indicative of risk of malnutrition and a score greater than 24 indicates a good nutritional state [72]. MNA requires approximately 10 minutes, and that is one of the main reasons of success, as it is used in a large number of studies.

SGA is based on a standardized questionnaire that includes medical history (variations before treatment calorie, weight loss, gastrointestinal symptoms, and functional capacity) and goals (signs of malnutrition with the presence of edema and/or changing the dial fat and muscle) [73].

## 7. Conclusions

Nutritional aspects in inflammatory bowel disease are particularly relevant as they could potently contribute to disease morbidity and mobility. It can be argued that a poor nutritional status, selective malnutrition or sarcopenia, can be associated with poor clinical outcomes, response to therapy, and quality of life. For this reason, a multidisciplinary assessment of IBD is to be sustained, making closer gastroenterological assessment and nutritional assessment in a broader and harmonically defined picture, where instruments, clinical mind, and evaluation come closer.

## Figures and Tables

**Table 1:** Micronutrient deficiencies in IBD.

Micronutrient	Physiopathology	Main symptoms of deficiency	Diagnosis
Iron	Chronic blood loss Impaired iron metabolism Inadequate intake	Anemia, fatigue, sleeping disorders, restless legs syndrome, attention deficit, discontentment, agitation, and female infertility	Transferrin sat <16% and serum ferritin <30 ng/mL

Calcium	Inadequate dietary intake Decreased intestinal/renal absorption	Decreased bone density, hyperparathyroidism, hypertension, and muscle spasm	Bone density scan Serum calcium < 8.5 mg/dl

Selenium	Not fully understood	Cardiomyopathy and cartilage degeneration	Serum selenium <70 *μ*g/L

Zinc	Chronic diarrhea Malabsorption	Poor wound healing	Serum zinc <75 *μ*g/mL

Magnesium	Chronic diarrhea Inadequate dietary intake	Fatigue	Serum magnesium <1.41 mEq/L

Vitamin B9	Inadequate dietary intake Malabsorption Medications (MTX)	Megaloblastic anemia, modestly increased risk of colonic dysplasia, and hyperhomocysteinemia	Serum folate < 2.5 ng/mL

Vitamin B_12_	History of ileal/ileocolonic resection	Megaloblastic anemia and peripheral neuropathy	Serum B_12_ < 200 pg/mL

Vitamin D	Inadequate dietary intake Malabsorption	Abnormal bone metabolism	Serum 25OHD (<15 ng/mL deficiency, <20 ng/mL insufficiency, and >30 ng/mL optimum)

Vitamin A	Inadequate dietary intake Malabsorption	Poor wound healing, night blindness, and xeropthalmia	Serum retinol <30 *μ*g/dL

Vitamin K	Inadequate dietary intake Malabsorption Use of antibiotics	Abnormal bone metabolism	Serum phylloquinone <1.1 ng/mL PT/INR
